# Relationship between Diet and Non-alcoholic Fatty Liver Disease: A Review Article

**Published:** 2017-08

**Authors:** Parvin MIRMIRAN, Zeynab AMIRHAMIDI, Hanieh-Sadat EJTAHED, Zahra BAHADORAN, Fereidoun AZIZI

**Affiliations:** 1. Nutrition and Endocrine Research Center, Research Institute for Endocrine Sciences, Shahid Beheshti University of Medical Sciences, Tehran, Iran; 2. Endocrine Research Center, Research Institute for Endocrine Sciences, Shahid Beheshti University of Medical Sciences, Tehran, Iran

**Keywords:** Non-alcoholic fatty liver disease, Food groups, Dietary patterns

## Abstract

**Background:**

Diet plays a key role in the development of non-alcoholic fatty liver disease (NAFLD). The aim of this study was to review systematically observational studies available regarding the relationship between food intakes and NAFLD.

**Methods:**

We searched Scopus, PubMed, and Cochrane Library databases to identify English observational studies on food groups, dietary patterns, and NAFLD. Cross-sectional, case-control and cohort studies were selected and then duplication, topic, type of study, study population, variables examined and quality of data reporting of the articles were evaluated.

**Results:**

We identified 2128 studies in the initial search, of which 33 were reviewed in full text and 7 articles were included in this systematic review. Intakes of red meat, fats, and sweets were high whereas consumption of whole grains, fruits and vegetables were less in NAFLD patients. Moreover, there was a positive association between the Western dietary pattern and the risk of NAFLD, while adherence to the Mediterranean diet was significantly associated with the severity of hepatic steatosis.

**Conclusion:**

Generally, different food group intakes and dietary patterns are associated with the progression of NAFLD and its risk factors. Because of the many limitations of available studies reviewed on this topic, more prospective studies are suggested.

## Introduction

Nonalcoholic fatty liver disease (NAFLD), characterized by increased fat accumulation in the hepatocytes of the liver parenchyma, is today one of the most common causes of chronic liver disease worldwide (1–4). NAFLD affects 20%–40% of population in Western countries and 5%–40% of general populations in Asia-Pacific region (5). The increasing prevalence of NAFLD parallels the rise of obesity, type 2 diabetes mellitus, and components of the metabolic syndrome, particularly insulin resistance (5–8). Development of NAFLD is considered to be through a “two hit” process. The first “hit” includes excessive fat accumulation in liver cells and insulin resistance. Oxidative stress and pro-inflammatory cytokines play roles in the second “hit” (5, 9).

The disease encompasses a wide spectrum of conditions ranging from simple steatosis to non-alcoholic steatohepatitis (NASH), which can progress to fibrosis, cirrhosis and hepatic carcinoma (5, 7, 10). Moreover, NAFLD patients are at increased risk of cardiovascular disease and chronic kidney disease (6, 11). Diet has a key role in the development of NAFLD. Genetic and positive energy balance have important impacts on the first “hit” and diet composition affects the second “hit” and the severity of NAFLD (8, 12, 13) emphasizing the criticality of management and control of NAFLD.

Several studies have reported that excessive consumption of carbohydrates, especially refined carbohydrates, fats, saturated fats in particular, and protein from meat can cause NAFLD (14–18). Besides, higher intakes of soft drinks and meat are associated with NAFLD in adults (14). To date lifestyle changes and dietary modifications are the main established treatment guidelines for NAFLD (3, 4, 19, 20). Lifestyle modifications involve weight loss and increasing physical activity (3, 8, 21). Weight loss by following a calorie-restricted diet and increasing physical activity can improve steatosis, dyslipidemia, insulin resistance, and cardiovascular risk and reduce hepatic inflammation and hepatocellular injury (2–4, 21). Moreover, all patients with NAFLD are advised to adhere to several dietary recommendations such as avoiding simple carbohydrates, saturated fats, and sweetened drinks and consuming diets rich in fruits and vegetables (3, 4, 13).

Although there are studies examined the relationship between NAFLD and food intakes, no systematic review has been conducted to summarize the findings. Thus, the objective of this study was to review systematically observational studies available on the relationship between food groups, dietary patterns, and non-alcoholic fatty liver disease.

## Methods

### Data sources

To identify relevant studies, a systematic search strategy of PubMed, Scopus, and Cochrane Library was conducted using queries including the keywords “non-alcoholic fatty liver disease”, “hepatic steatosis”, “steatohepatitis”, and “NAFLD”, “nutrition”, “dietary pattern”, “food groups”, “fruits”, “vegetables”, “dairy”, “grains”, “fats”, and “meats”. All searches were limited to studies published in English.

### Eligibility criteria

Studies with design of cohort, case-control, and cross-sectional setting have evaluated the association between dietary patterns, food group intakes and NAFLD were initially included in the review.

### Study Selection

Two researchers performed study selection independently. First, the titles and abstracts of all retrieved studies were screened. Then, the full texts of potentially eligible studies were reviewed before definitive inclusion. Disagreements between the two authors were resolved by consensus.

### Data extraction

Relevant data were extracted from studies. The data form included the following information: first author’s name and year of publication, country and study design, number of participants, range of follow-up, demographic characteristics of participants, methods of dietary intake assessment and NAFLD diagnosis, dietary factors evaluated, statistics for association between dietary patterns, food group intakes and NAFLD.

### Quality assessment

We used the “Strengthening the Reporting of Observational Studies in Epidemiology” (STROBE) checklist to assess the quality of included studies. Scoring definitions were derived from the explanation and elaboration document (22). The quality of reporting of the 33 studies included in full-text step was assessed by the two authors and five studies which had the quality scores < 50% were excluded.

## Results

**Fig. 1:**
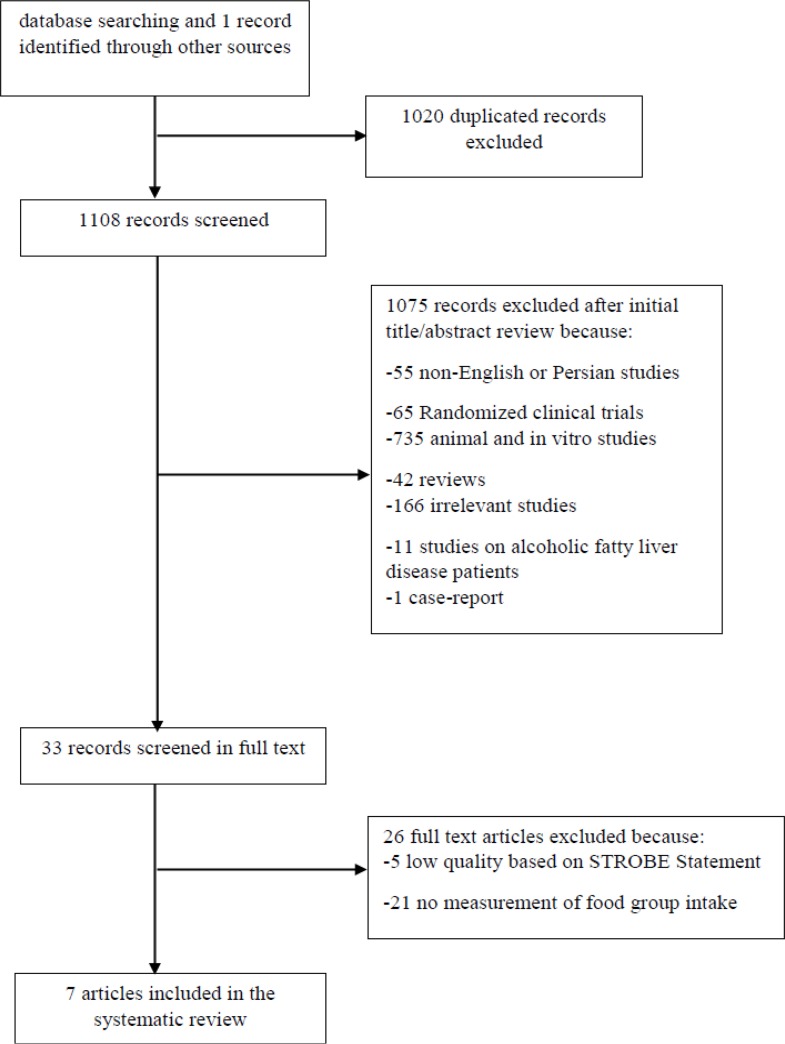
Flowchart of the search and selection process for articles included in the systematic review

The initial search identified 2128 articles, of which 1020 records were duplicated and 1108 were considered eligible for further evaluation. Of these, 1075 were excluded because they did not fulfill the inclusion criteria; 33 articles were reviewed in full text and eventually, a total of 7 articles (two cross-sectional studies, four case-control studies, and one cohort study) published between 2010 and 2013 were selected (12, 23–28) (Fig. 1).

The studies were from Brazil, America, Greece, Iran, China, and Australia. The food frequency questionnaire (FFQ) was the most common dietary assessment tool used in the studies (n=6) (12, 23, 24, 26–28), whereas the dietary record was only used in one study (25). The number of food items was ranged 17 to 212, in different versions of the FFQ; dietary assessment was included information of dietary intakes over the past year and the food groups, assessed in these studies, was included bread and grains, meat and beans, fruits, vegetables, milk and dairy and fats and oils. Abdominal ultrasonography was used in nearly all of the studies for NAFLD diagnosis. According to the STROBE criteria, the quality of the selected articles was ranged 57%–80%. In case-control studies, participants were matched in terms of sex, age, and body mass index. In the cohort study, data was adjusted for the Western and the healthy dietary pattern, sex, misreporting, income, watching TV and physical activity at age 14 yr. Summary of characteristics and findings of these studies were shown in Table 1.

**Table 1:** Summary of characteristics and findings from reviewed studies

**Author**	**Study design and Follow-up**	**Study population**	**Dietary intake assessment and NAFLD diagnosis**	**Factors studied**	**Findings**
Ferolla et al. 2013(23)	Cross-sectional	96 men and women, median age: 53 yr, Brazil	80-item Semi-quantitative food-frequency questionnaire and 24-hour food recall/ Abdominal ultrasound	All food groups (bread and grains, meat and beans, fruits, vegetables, milk and dairy, fats and sweets)	A significantly higher intake of meats, fats, sugars, legumes and vegetables in the patients compared with the recommendations (*P*<0.05 )
					A significantly lower consumption of cereals, fruits and dairy products in patients compared to the recommendations (*P*<0.05 )
Kim et al. 2010(24)	Cross-sectional	74 men and women, mean age: 52.5, America	13-item food-frequency questionnaire	All food groups (bread and grains, meat and beans, fruits, vegetables, milk and dairy, fats and sweets)	Higher calories intake from meat and bean in NAFLD patients compared with HBV and HCV (2424.8 vs. 2251 and 2057, respectively, *P*=0.316 )
					Higher calories intake from milk in NAFLD patients compared with HBV (845 vs. 620.6, *P*=0.0137 )
					Lower calories intake from fruits in NAFLD patients compared with HBV and HCV (715.6 vs. 943.4 and 931.2, respectively, *P*=0.0112 )
HashemiKani et al. 2013(25)	Case-control	NAFLD patient: 100 men and women, mean age: 37.9	Three dietary records (one weekend and two week days)/ Sonography	Different food groups (grains, meat, fruits, vegetables, dairy, poultry and fish) and dietary indices (Dietary Energy Density (DED), Dietary Diversity Score (DDS), Healthy Eating Index (HEI), Mean Adequacy Ratio MAR))	Higher intake of refined grains in NAFLD patients compared with controls (378.1 vs. 267.1, P=0.001)
					Lower intake of whole grains and vegetables in NAFLD patients compared with controls (31.3 vs. 61, P=0.001; 293.7 vs. 359.9, *P*=0.01, respectively )
		Control: 100 men and women, mean age: 37.9, Iran			
					Lower values of HEI, DDS and MAR in NAFLD patients compared with controls (53.3 vs. 63.9, *P*=0.01; 5.9 vs. 7.7, *P*=0.06; 10.6 vs. 14.1, *P*=0.03, respectively )
					Higher amount of DED in NAFLD patients compared with controls (1.7 vs. 1.1, *P*=0.05) Marginally significant association between all the dietary quality indices and NAFLD (P_trend_≤0.09 )
Shi et al. 2012(26)	Case-control	NAFLD patient:200 men and women aged 20–90	17-item food-frequency questionnaire/ Abdominal ultra-sound	Different food groups (rice and wheat, coarse cereals, potatoes, vegetables, fruits, meat, dairy and nuts)	Lower intake of coarse cereals, potatoes, vegetables, fruits and milk in NAFLD patients compared with control group (P<0.05)
		Control: 200 men and women aged 20–90, China			Higher intake of red meat, viscera, candies and pastries and cooking oil in NAFLD patients compared with control group (P<0.05)
					A significant association between frequent dessert consumption and NAFLD (OR 4.524; 95% CI 1.415–14.462; P=0.011),
					A significant association between frequent salty food consumption and NAFLD (OR 2.333; 95%CI 1.152–4.724; P=0.019)
					A significant association between frequent spicy food consumption and NAFLD(OR 2.192; 95%CI 1.030–4.664; P=0.042)
Magalhaes et al. 2013(27)	Case-control	NAFLD patient: 24 women aged 20–50 Control: 36 women aged 20–50, Brazil	13-item qualitative food frequency questionnaire/ Abdominal ultrasound	Different food groups (vegetables, fruits, meat and beans, eggs, dairy, sweets, soft drinks, chocolates and fried foods)	A non-significantly higher intake of meats, fruits, beans, sweets and chocolates in NAFLD patients compared with controls
					A non-significantly lower intake of dairy products, eggs, vegetables, soft drinks and fried foods in NAFLD patients compared with controls
Kontogianni et al. 2013(12)	Case-control	NAFLD patient: 58 men and women aged 18–65	60-item semi quantitative food frequency questionnaire/ Ultra-sonography	Mediterranean Diet Score	Negative association between Mediterranean diet score and logHOMA-IR (standardized beta coefficient= −0.303, P=0.005)
		Control: 58 men and women aged 18–65, Greece			Negative correlation between adherence to the Mediterranean diet and steatosis (Rho= −0.52, P=0.006)
Oddy et al. 2013(28)	Cohort/ 3 yr	995, 14 year-old men and women, Australia	212-item semi quantitative food frequency questionnaire/ Ultra-sonography	Different food groups (soft drinks, full-fat dairy, refined grains, red meat, takeaway foods, potato, confectionary, processed meats, cakes and biscuits, fried chips, sauces and dressings, crisps)	

### Food groups intake and non-alcoholic fatty liver disease

Five studies including two cross-sectional (23, 24), two case-control (25, 26) and one cohort (28), examined the relationship between food group intakes and NAFLD; results of four of these showed that grains consumption in patients with NAFLD was lower compared to healthy subjects or the recommended values.

In a cross-sectional study performed in Brazil (23) on 96 patients with NAFLD, the average number of consumed cereals was lower than the recommended number in the Brazilians Dietary Guideline (0.3 *vs.* 2.1 meals/day, *P*<0.01). A cross-sectional study from the USA (24), of 74 patients with NAFLD, Hepatitis C Virus (HCV) and Hepatitis B Virus (HBV), indicated that NAFLD patients had lower weekly serving cereals and grains scores than HBV patients and higher than those of the HCV group (5.11 *vs.* 7.63 and 5.11 *vs.* 4.88, *P*<0.01, respectively). A case-control study was performed on 100 NAFLD patients and 100 healthy subjects in Iran (25); whole grain intakes in the patients was lower than in controls (31.3 *vs.* 61 g/d, *P*=0.001) and refined-grain intakes in cases was higher than in healthy subjects (378.1 *vs.* 267.1 g/d, *P*=0.001). A case-control study (26) in China was conducted on 200 non-alcoholic fatty liver disease patients and 200 controls. The average rice and wheat in-take in the patients were non-significantly higher than in controls. Whereas, the average consumption of coarse cereals in NAFLD patients was lower than patients with healthy subjects (15.4 *vs.* 23.4, *P*<0.05).

However, a cohort study conducted (28) on 995, 14-year-old healthy subjects after 3 yr follow-up at 17 yr, the association between refined grains and NAFLD was not significant. Of these five studies, in the four studies (23–26) consumption of red meat and its products in NAFLD patients was higher than in controls or recommended values. Intake of meat in the patients was significantly higher than the guide (*P*=0.003) (23). In addition, patients with NAFLD were consumed more calories of meat and bean than HCV and HBV (P=0.0316) (24). Red meat and viscera consumption were higher in NAFLD patients than in controls (150.4 *vs.* 97.4 g/d and 9.7 *vs.* 3.4 g/d, *P*<0.05, respectively) (26). In addition, non-significantly more consumption of red meat in the patients were compared with the healthy subjects (25) and in the study (28) this examined association was non-significant.

The findings of this systematic review regarding dairy intake in NAFLD patients was inconsistent. NAFLD patients consumed more calories of dairy products compared with HBV patients (845.2 *vs.* 620.6 calories, P=0.0137) (24). The average number of meal consumed with dairy in NAFLD patients was less than recommended in the Dietary Guide for Brazilians (0.4 *vs.* 1.5 meals/day, *P*<0.01) (23). However, milk and derived products intake in NAFLD patients were lower than the controls (137.6 *vs.* 194.1 ml/day, *P*<0.05) (26). Besides, no difference was reported in dairy intake between cases (25) and controls and non-significant association were shown between full-fat dairy intake and NAFLD (28).

Based on the findings of the four studies of five in this category (23–26), fruits intake in patients with NAFLD was lower than healthy subjects were or recommended amounts. The average number of meal consumed with fruits in NAFLD patients was less than recommended amounts (0.9 *vs.* 1.5 meals/day, *P*<0.01) (23). NAFLD patients consumed fewer calories of fruits compared with HBV and HCV groups (715 *vs.* 943 and 715 *vs.* 931 calories, *P*=0.0112) (24). There was non-significantly lower consumption of fruits in the patients compared with the controls (25). Intake of fruits was reported in the patients lower than in healthy subjects (89.4 *vs.* 109.3 g/day, *P*<0.05) (26).

In three of these five studies (24–26), there was a low intake of vegetables among NAFLD patients. NAFLD patients had lower score of weekly serving vegetable consumption compared with HCV and HBV groups (14.55 *vs.* 15.91 and 14.55 *vs.* 18.17, *P*=0.04, respectively) (24). The patients had less intake of vegetables than the healthy subjects (293 *vs.* 360 g/day, *P*=0.01) (25). However, lower vegetables intake in NAFLD patients compared with the controls (435 *vs.* 480 g/day, *P*<0.05) (26). However, number of meals consumed with vegetables in the patients was higher than recommended in the Dietary Guide for Brazilians (1.6 *vs.* 1.5 meals/day, *P*=0.01) (23).

The three of these studies showed higher consumption of sugars and fats in NAFLD patients than in controls or recommended values. Number of meals consumed with fats and sugars in the patients was higher than recommended in the Dietary Guide (2.4 *vs.* 0.5 and 1.0 *vs.* 0.5 meals/day, *P*<0.01, respectively) (23). Higher score of weekly serving fats, oil and sweets consumption was reported in NAFLD patients compared with HBV subjects (11.6 *vs.* 7.4, *P*<0.01) (24). Consumption of cooking oil, candies and pastries in the patients were higher than in the controls (47.2 *vs.* 30.7 and 11.6 *vs.* 7.4, *P*<0.05, respectively) (26). Frequent consumption of dessert (OR=4.5; 95% CI=1.4–14.4; *P*=0.01), salty food (OR=2.3; 95% CI 1.15–4.72; *P*=0.019) and spicy food (OR=2.19; 95% CI=1.03–4.66; *P*=0.042) were associated with NAFLD. Higher consumption of soft drinks was showed in NAFLD patients (28). Moreover, in this study, those in the highest quartile for soft drinks had 93% greater risk of NAFLD (OR=1.93; 95% CI=1.04–3.56; *P*<0.05) compared with those in the lowest quartile. Besides, there was a high risk of NAFLD in those in the highest quartile for sauces and dressings (OR=1.95; 95% CI=1.12–3.41; *P*<0.05) compared with those in the lowest quartile. In addition, there was a significant association between increasing quartiles of sauces and dressings and the risk of NAFLD (*P*_trend_=0.003).

In one of the studies included in this systematic review (27), on 24 NAFLD patients and 36 controls, frequency of habitual food intake including dairy products, meats and beans, vegetables, fruits, grains, sweets and chocolates, soft drinks and fried foods were assessed. The results showed no significant differences between NAFLD patients and controls for frequency consumption of these food groups.

### Different dietary patterns and non-alcoholic fatty liver disease

In one study (12), a case-control study was conducted on 58 patients with NAFLD and 58 healthy subjects and was estimated adherence to the Mediterranean diet with Mediterranean Diet Score (Med Diet Score). To determine this score, they assessed the intake of 9 food groups: not-refined starchy food, potatoes, fruits, vegetables, legumes, fish, meat and meat products, poultry and full-fat dairy products and also olive oil and alcoholic beverages. Med Diet Score did not differ significantly between cases and controls. Adherence to the Mediterranean diet was not associated with the likelihood of having NAFLD but this Score was significantly associated with the severity of steatosis (Rho=−0.52, *P*=0.006). Moreover, one unit increase in the Med Diet Score was associated with 36% lower likelihood of having non-alcoholic steatohepatitis (OR=0.64, 95%CI: 0.45–0.92, *P*=0.02) and Med Diet Score was negatively associated with log HOMA-IR (standardized beta coefficient: −0.303, *P*=0.005).

The participants’ diets were assessed with an evaluated semi-quantitative FFQ at baseline, at age 14 yr, and then after 3 yr follow-up, at 17 yr, their non-alcoholic fatty liver disease status was examined by ultrasonography (28). Healthy and Western dietary patterns were identified and all participants received a z-score for these two patterns. Of the 995 individuals who completed a food frequency questionnaire at age 14 yr, 151 were diagnosed with NAFLD at age 17 yr (15.2%) and of those who had the disease at age of 17 yr, 54.1% were overweight or obese at 14 yr age. Higher score for the Western dietary pattern, characterized by high intakes of takeaway foods, red meats, processed meats, full-fat dairy products, fried potatoes, refined cereals, cakes and biscuits and confectionery, soft drinks, crisps, sauces and dressings, was positively associated with the odds of non-alcoholic liver disease at 17 yr (OR 1.59; 95%CI 1.17–2.14; *P*=0.003). In addition, there was a significant association between increasing quartiles of the Western dietary pattern scores and odds of NAFLD (P _trend_=0.03). Moreover, those in the highest quartile of the Western dietary pattern had 2.6 times greater odds of NAFLD compared with those in the lowest quartile (OR=2.64; 95% CI: 1.34–5.18, *P*=0.005). However, after adjustment for BMI at age of 14 yr this association was non-significant, indicating that the association between NAFLD and the Western dietary pattern is likely acting through an obesity pathway. In this study, a protective association between the healthy dietary pattern, positively related to whole grains, fruits, vegetables, legumes, fish and fiber and was inversely correlated with energy from total fat, saturated fat and refined sugar, and the disease was not observed but in a subgroup of participants with central obesity at age of 17 yr (n=191), a healthy dietary pattern was significantly associated with a reduced risk of NAFLD (OR=0.63; 95%CI: 0.41–0.96, *P*=0.033).

### Dietary quality indices and non-alcoholic fatty liver disease

A case-control study was investigated the relation between dietary quality indices and NAFLD (25). In this study, four dietary quality indices were assessed: Dietary Energy Density (DED), Dietary Diversity Score (DDS), Healthy Eating Index (HEI) and Mean Adequacy Ratio (MAR). DED calculates energy density from food only as energy (kcal)/weight of food (g) excluding non-energetic beverages. DDS and HEI evaluate the intake of five food groups, bread-grains, vegetables, fruits, meats and dairy and MAR is the mean adequacy ratio of daily individual intakes to standard recommended amounts for subject’s sex and age category. All of these indices were associated with NAFLD at a marginally significant level. The patients had lower values of HEI, DDS, and MAR and higher amount of DED than healthy subjects did. Compared to the fourth quartile, participants who were in the first quartile of DED had a 47% more chance of having NAFLD (OR=0.53, 95% CI=0.19–0.89). Moreover, the chance for having NAFLD decreased (P_trend_=0.07, P _trend_=0.09, and P _trend_=0.05; respectively) across increasing intakes of HEI, DDS and MAR; the likelihood of developing NAFLD also increased (P _trend_=0.05) across increasing trends of DED.

## Discussion

This is the first systematic review regarding the association between food intakes and non-alcoholic fatty liver disease. The results of our review suggest that there are relationships between various food groups intake, diets, and NAFLD but not clear and conclusive ones and NAFLD patients consume difference amounts of various food groups. The reasons for these differences and uncertainties are different study designs (12, 23–26), small sample size (12, 25, 27), no control of confounder variables such as anthropometric factors and socioeconomic status (23, 25–27), the diagnostic method of NAFLD (12, 23, 25–27), bias caused by FFQ (12, 23, 24, 26) and no control group or unhealthy controls (23–25, 27). Therefore, it is necessary to give special attention to these factors in future studies in this field.

We can discuss the relationship between diet and NAFLD in different perspectives. Some of them studied the association between food group in-takes and NAFLD. Red meat and its products, fats and oils and sugars and sweets intake were higher and whole grains, fruits and vegetables intake were lower in the patients than in the controls or recommended amounts. Generally, review of studies has shown dairy consumption can reduce the risk of obesity, particularly abdominal obesity, diabetes and hypertension, which are NAFLD risk factors (29, 30). However, the results of included articles in this review were inconsistent because their quality was influenced by some major limitations like different study design (23, 24, 26), no control group (24) or obese participants (23, 26). Therefore, more prospective studies are needed in this field.

In most of the reviewed studies, fruits and vegetables consumption were lower in NAFLD patients compared with healthy subjects or recommended amounts. The protective effects of high intake of fruits and vegetables on NAFLD prevention are due to a high content of fiber, phytochemicals, and antioxidants in these food groups. Phytochemicals and antioxidants are anti-inflammatory compounds and can prevent developing hepatic steatosis. Moreover, fiber plays an important role in maintaining blood glucose, insulin and free fatty acids at a constant level, in patients with NAFLD (31, 32). This result can be the best explain for the reason of lower hepatic steatosis in individuals who adhere to the Mediterranean diet because there are high amounts of fruits and vegetables in this dietary pattern (12, 33, 34).

Moreover, most of the studies in this systematic review showed higher consumption of red meat in NAFLD patients. Saturated fatty acids in red meat increase trans-10, cis-12 conjugated linoleic acid in liver cells (35) and so can promote endoplasmic reticulum stress and apoptosis (36), involved in the pathogenesis of NAFLD.

**Fig. 2:**
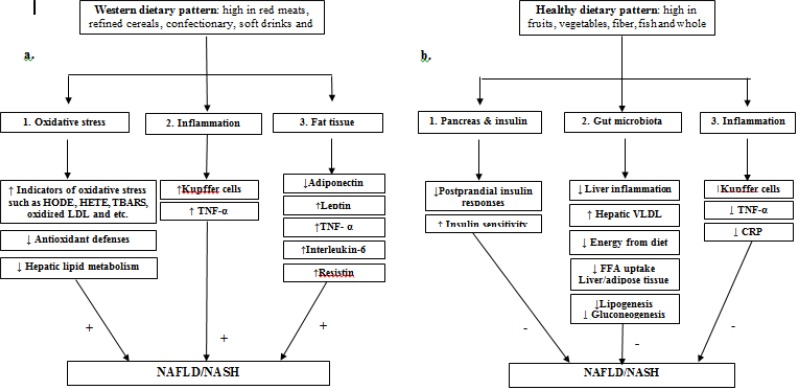
Effects of western and healthy dietary patterns on the development of nonalcoholic fatty liver disease: a. western dietary pattern can cause NAFLD through several ways: 1) by ↑ HODE (hydroxyoctadecadienoic acids), HETE (hydroxyeicosatetraenoic acids), TBARS (thiobarbituric acid-reactive substances) and oxidized LDL (lipid oxidation products), so increase in oxidative stress, 2) by ↑ inflammation mediated by Kupffer cells and TNF-α, and 3) by alteration in secretion of adipokines from fat tissues for example: reduction in adiponectin and increase in leptin, TNF-α, interleukin-6 and resisting. b. Healthy dietary pattern can provide protection towards development of NAFLD through: 1)by effect on pancreas and so decrease in postprandial insulin responses and increase in insulin sensitivity, 2) by affecting the growth of gut bacteria (suppression in the growth of pathogenic bacteria and stimulation in beneficial ones)leading to↓ FFA uptake in Liver/adipose tissue, ↓ lipogenesis, ↓ gluconeogenesis, ↓ energy harvest from diet, ↑ VLDL secretion from liver and ↓ liver inflammation, and 3)by ↓ Kupffer cells, TNF-α and CRP and so reduction in inflammation.

Some other studies in this review, evaluated the association between diet and non-alcoholic fatty liver disease, from the perspective of dietary patterns (12, 28). Review of these studies showed the Western dietary pattern was prospectively associated with NAFLD. In fact, common foods in the Western dietary pattern including processed sugars and fat, white bread, refined grains, soft drinks, and confectionary, by provision of excess calories and large amounts of sugars such as fructose, can cause rapidly increased postprandial plasma glucose and insulin levels. (28, 37–39). Several studies showed a significant association between the Western dietary pattern and obesity (40, 41). Obese individuals were followed a Western dietary pattern had a greater rate of *de novo* lipogenesis, one of the causes of NAFLD, compared with lean subjects when fed the same diet (42).. Based on these findings and other studies regarding the pathogenesis and risk factors of NAFLD and different dietary patterns (43–52) we can suggest Fig. 2.

Review of the studies (12, 53, 54) also showed adherence to the Mediterranean diet because of the high content of fruits and vegetables, is inversely associated with insulin resistance. Moreover, this diet can provide protective effects on metabolic syndrome and its components and improves insulin sensitivity and hepatic steatosis in patients with NAFLD (55).

The third category of included studies in the review examined the association between dietary quality indices and NAFLD (25). To determine these indices, researchers assess the total intakes of all food groups and then compare them with standard recommended amounts. DED, HEI, MAR, and DDS could be appropriate indicators to assess the dietary quality in them. The observed score of these indices among NAFLD patients, lower score of HEI, DDS and MAR and higher score of DED, are reasonable because the NAFLD patients consume lower fruits, vegetables, whole grains, fiber and antioxidants and higher carbohydrate, especially simple carbohydrate, refined grains, and unsuitable fat (17, 18, 33, 56).

The remarkable point in this regard is the importance of non-dietary factors, particularly genetic, in the pathogenesis of non-alcoholic fatty liver disease (55). Positive energy balance, involved in the first hit of the disease, has different effects in different individuals. This issue is also supported by studies indicating different likelihood of NAFLD among different races (57). In fact, these non-dietary factors may be responsible for some of the inconsistent findings regarding the role of diet composition in the pathogenesis and development of NAFLD (12).

## Conclusion

Different food group intakes and dietary patterns are associated with NAFLD and its risk factors such as obesity, glucose intolerance, type 2 diabetes mellitus and insulin resistance. However, the generalizability of these results is limited because the design of the most of reviewed studies was cross-sectional or case-control which cannot establish causal relations. Therefore, we suggest doing more prospective studies in future to clarify these associations in a prospective view.

## Ethical considerations

Ethical issues (Including plagiarism, informed consent, misconduct, data fabrication and/or falsification, double publication and/or submission, redundancy, etc.) have been completely observed by the authors.

## References

[1] Anderson EL Howe LD Fraser A (2015). Childhood Energy Intake Is Associated with Nonalcoholic Fatty Liver Disease in Adolescents. J Nutr, 145:983–9. 25788585 10.3945/jn.114.208397 PMC4410498

[2] Loomba R Cortez-Pinto H (2015). Exercise and improvement of NAFLD: Practical recommendations. J Hepatol, 63:10–2. 25863525 10.1016/j.jhep.2015.03.009

[3] Nseir W Hellou E Assy N (2014). Role of diet and lifestyle changes in nonalcoholic fatty liver disease. World J Gastroenterol, 20:9338–44. 25071328 10.3748/wjg.v20.i28.9338 PMC4110565

[4] Sofi F Casini A (2014). Mediterranean diet and non-alcoholic fatty liver disease: New therapeutic option around the corner? World J Gastroenterol, 20:7339–46. 24966604 10.3748/wjg.v20.i23.7339 PMC4064079

[5] Abd El-Kader SM El-Den Ashmawy EM (2015). Non-alcoholic fatty liver disease: The diagnosis and management. World J Hepatol, 7:846–58. 25937862 10.4254/wjh.v7.i6.846 PMC4411527

[6] Al Rifai M Silverman MG Nasir K (2015). The association of nonalcoholic fatty liver disease, obesity, and metabolic syndrome, with systemic inflammation and subclinical atherosclerosis: the Multi-Ethnic Study of Atherosclerosis (MESA). Atherosclerosis, 239:629–33. 25683387 10.1016/j.atherosclerosis.2015.02.011 PMC4406399

[7] Arab JP Candia R Zapata R (2014). Management of nonalcoholic fatty liver disease: an evidence-based clinical practice review. World J Gastroenterol, 20:12182–201. 25232252 10.3748/wjg.v20.i34.12182 PMC4161803

[8] Koppe SW (2014). Obesity and the liver: nonalcoholic fatty liver disease. Transl Res, 164:312–22. 25028077 10.1016/j.trsl.2014.06.008

[9] Fabbrini E Sullivan S Klein S (2010). Obesity and nonalcoholic fatty liver disease: biochemical, metabolic, and clinical implications. Hepatology, 51:679–89. 20041406 10.1002/hep.23280 PMC3575093

[10] Aguirre L Portillo MP Hijona E Bujanda L (2014). Effects of resveratrol and other polyphenols in hepatic steatosis. World J Gastroenterol, 20:7366–80. 24966607 10.3748/wjg.v20.i23.7366 PMC4064082

[11] Bonora E Targher G (2012). Increased risk of cardiovascular disease and chronic kidney disease in NAFLD. Nat Rev Gastroenterol Hepatol, 9:372–81. 22565095 10.1038/nrgastro.2012.79

[12] Kontogianni MD Tileli N Margariti A (2014). Adherence to the Mediterranean diet is associated with the severity of non-alcoholic fatty liver disease. Clin Nutr, 33:678–83. 24064253 10.1016/j.clnu.2013.08.014

[13] Dyson J Day C (2014). Treatment of non-alcoholic fatty liver disease. Dig Dis, 32:597–604. 25034293 10.1159/000360511

[14] Zelber-Sagi S Nitzan-Kaluski D Goldsmith R (2007). Long term nutritional intake and the risk for non-alcoholic fatty liver disease (NAFLD): A population based study. J Hepatol, 47:711–717. 17850914 10.1016/j.jhep.2007.06.020

[15] Cortez-Pinto H Jesus L Barros H Lopes C Moura MC Camilo ME (2006). How different is the dietary pattern in non-alcoholic steatohepatitis patients? Clin Nutr, 25:816–23. 16677739 10.1016/j.clnu.2006.01.027

[16] Musso G Gambino R De Michieli F (2003). Dietary habits and their relations to insulin resistance and postprandial lipemia in nonalcoholic steatohepatitis. Hepatology, 37:909–16. 12668986 10.1053/jhep.2003.50132

[17] Agius L (2013). High-carbohydrate diets induce hepatic insulin resistance to protect the liver from substrate overload. Biochem Pharmacol, 85:306–12. 23022226 10.1016/j.bcp.2012.09.019

[18] Colak Y Tuncer I Senates E (2012). Nonalcoholic fatty liver disease: A nutritional approach. Metab Syndr Relat Disord, 10:161–166. 22394108 10.1089/met.2011.0145

[19] Martin-Dominguez V Gonzalez-Casas R Mendoza-Jimenez-Ridruejo J (2013). Pathogenesis, diagnosis and treatment of non-alcoholic fatty liver disease. Rev Esp Enferm Dig, 105:409–20. 24206551 10.4321/s1130-01082013000700006

[20] Kaser S Ebenbichler CF Tilg H (2010). Pharmacological and non-pharmacological treatment of non-alcoholic fatty liver disease. Int J Clin Pract, 64:968–83. 20584230 10.1111/j.1742-1241.2009.02327.x

[21] Montesi L Caselli C Centis E (2014). Physical activity support or weight loss counseling for nonalcoholic fatty liver disease? World J Gastroenterol, 20:10128–10136. 25110440 10.3748/wjg.v20.i29.10128 PMC4123342

[22] Vandenbroucke JP von Elm E Altman DG (2007). Strengthening the Reporting of Observational Studies in Epidemiology (STROBE): explanation and elaboration. Epidemiology, 18:805–35. 18049195 10.1097/EDE.0b013e3181577511

[23] Ferolla SM Ferrari TC Lima ML (2013). Dietary patterns in Brazilian patients with nonalcoholic fatty liver disease: a cross-sectional study. Clinics (Sao Paulo), 68:11–7. 23420151 10.6061/clinics/2013(01)OA03 PMC3552450

[24] Kim CH Kallman JB Bai C (2010). Nutritional assessments of patients with non-alcoholic fatty liver disease. Obes Surg, 20:154–60. 18560947 10.1007/s11695-008-9549-0

[25] Hashemi kani A Alavian SM Esmaillzadeh A Adibi P Azadbakht L (2013). Dietary Quality Indices and Biochemical Parameters Among Patients With Non Alcoholic Fatty Liver Disease (NAFLD). Hepat Mon, 13:e10943. 24065998 10.5812/hepatmon.10943 PMC3776150

[26] Shi L Liu ZW Li Y (2012). The prevalence of nonalcoholic fatty liver disease and its association with lifestyle/dietary habits among university faculty and staff in Chengdu. Biomed Environ Sci, 25:383–91. 23026517 10.3967/0895-3988.2012.04.002

[27] Magalhaes GC Feitoza FM Moreira SB (2014). Hypoadiponectinaemia in nonalcoholic fatty liver disease obese women is associated with infrequent intake of dietary sucrose and fatty foods. J Hum Nutr Diet, 27 Suppl 2:301–12. 23790128 10.1111/jhn.12110

[28] Oddy WH Herbison CE Jacoby P (2013). The Western dietary pattern is prospectively associated with nonalcoholic fatty liver disease in adolescence. Am J Gastroenterol, 108:778–85. 23545714 10.1038/ajg.2013.95

[29] Jaffiol C (2008). [Milk and dairy products in the prevention and therapy of obesity, type 2 diabetes and metabolic syndrome]. Bull Acad Natl Med, 192:749–58. 19024947

[30] Bortolotti M Rudelle S Schneiter P (2008). Dairy calcium supplementation in overweight or obese persons: its effect on markers of fat metabolism. Am J Clin Nutr, 88:877–85. 18842771 10.1093/ajcn/88.4.877

[31] Anderson JW Randles KM Kendall CW Jenkins DJ (2004). Carbohydrate and fiber recommendations for individuals with diabetes: a quantitative assessment and meta-analysis of the evidence. J Am Coll Nutr, 23:5–17. 14963049 10.1080/07315724.2004.10719338

[32] Zivkovic AM German JB Sanyal AJ (2007). Comparative review of diets for the metabolic syndrome: implications for nonalcoholic fatty liver disease. Am J Clin Nutr, 86:285–300. 17684197 10.1093/ajcn/86.2.285

[33] Bullo M Lamuela-Raventos R Salas-Salvado J (2011). Mediterranean diet and oxidation: nuts and olive oil as important sources of fat and antioxidants. Curr Top Med Chem, 11:1797–810. 21506929 10.2174/156802611796235062

[34] Estruch R (2010). Anti-inflammatory effects of the Mediterranean diet: the experience of the PREDIMED study. Proc Nutr Soc, 69:333–40. 20515519 10.1017/S0029665110001539

[35] Wang D Wei Y Pagliassotti MJ (2006). Saturated fatty acids promote endoplasmic reticulum stress and liver injury in rats with hepatic steatosis. Endocrinology, 147:943–51. 16269465 10.1210/en.2005-0570

[36] Wei Y Wang D Pagliassotti M (2007). Saturated fatty acid-mediated endoplasmic reticulum stress and apoptosis are augmented by trans-10, cis-12-conjugated linoleic acid in liver cells. Mol Cell Biochem, 303:105–113. 17426927 10.1007/s11010-007-9461-2

[37] Dekker MJ Su Q Baker C (2010). Fructose: a highly lipogenic nutrient implicated in insulin resistance, hepatic steatosis, and the metabolic syndrome. Am J Physiol Endocrinol Metab, 299:E685–94. 20823452 10.1152/ajpendo.00283.2010

[38] Abid A Taha O Nseir W (2009). Soft drink consumption is associated with fatty liver disease independent of metabolic syndrome. J Hepatol, 51:918–24. 19765850 10.1016/j.jhep.2009.05.033

[39] Assy N Nasser G Kamayse I (2008). Soft drink consumption linked with fatty liver in the absence of traditional risk factors. Can J Gastroenterol, 22:811–6. 18925303 10.1155/2008/810961 PMC2661299

[40] Berkey CS Rockett HR Field AE (2000). Activity, dietary intake, and weight changes in a longitudinal study of preadolescent and adolescent boys and girls. Pediatrics, 105:E56. 10742377 10.1542/peds.105.4.e56

[41] Ritchie LD Spector P Stevens MJ (2007). Dietary patterns in adolescence are related to adiposity in young adulthood in black and white females. J Nutr, 137:399–406. 17237318 10.1093/jn/137.2.399

[42] Schwarz JM Linfoot P Dare D Aghajanian K (2003). Hepatic de novo lipogenesis in normoinsulinemic and hyperinsulinemic subjects consuming high-fat, low-carbohydrate and low-fat, high-carbohydrate isoenergetic diets. Am J Clin Nutr, 77:43–50. 12499321 10.1093/ajcn/77.1.43

[43] Sumida Y Niki E Naito Y Yoshikawa T (2013). Involvement of free radicals and oxidative stress in NAFLD/NASH. Free Radic Res, 47:869–80. 24004441 10.3109/10715762.2013.837577

[44] Rolo AP Teodoro JS Palmeira CM (2012). Role of oxidative stress in the pathogenesis of nonalcoholic steatohepatitis. Free Radic Biol Med, 52:59–69. 22064361 10.1016/j.freeradbiomed.2011.10.003

[45] Abenavoli L Peta V (2014). Role of adipokines and cytokines in non-alcoholic fatty liver disease. Rev Recent Clin Trials, 9:134–40. 25514909 10.2174/1574887109666141216102458

[46] Arslan N (2014). Obesity, fatty liver disease and intestinal microbiota. World J Gastroenterol, 20:16452–16463. 25469013 10.3748/wjg.v20.i44.16452 PMC4248188

[47] Wenfeng Z Yakun W Di M (2014). Kupffer cells: increasingly significant role in nonalcoholic fatty liver disease. Ann Hepatol, 13:489–95. 25152980

[48] Goel A Gupta M Aggarwal R (2014). Gut microbiota and liver disease. J Gastroenterol Hepatol, 29:1139–48. 24547986 10.1111/jgh.12556

[49] Abenavoli L Milic N Peta V (2014). Alimentary regimen in non-alcoholic fatty liver disease: Mediterranean diet. World J Gastroenterol, 20:16831–40. 25492997 10.3748/wjg.v20.i45.16831 PMC4258553

[50] Asrih M Jornayvaz FR (2013). Inflammation as a potential link between nonalcoholic fatty liver disease and insulin resistance. J Endocrinol, 218:R25–36. 23833274 10.1530/JOE-13-0201

[51] Kong LC Holmes BA Cotillard A (2014). Dietary patterns differently associate with inflammation and gut microbiota in overweight and obese subjects. PLoS One, 9:e109434. 25330000 10.1371/journal.pone.0109434 PMC4203727

[52] Casas R Sacanella E Estruch R (2014). The immune protective effect of the Mediterranean diet against chronic low-grade inflammatory diseases. Endocr Metab Immune Disord Drug Targets, 14:245–54. 25244229 10.2174/1871530314666140922153350 PMC4443792

[53] Kastorini CM Milionis HJ Esposito K (2011). The effect of Mediterranean diet on metabolic syndrome and its components: a meta-analysis of 50 studies and 534, 906 individuals. J Am Coll Cardiol, 57:1299–313. 21392646 10.1016/j.jacc.2010.09.073

[54] Puppala J Siddapuram SP Akka J Munshi A (2013). Genetics of nonalcoholic Fatty liver disease: an overview. J Genet Genomics, 40:15–22. 23357341 10.1016/j.jgg.2012.12.001

[55] Ryan MC Itsiopoulos C Thodis T (2013). The Mediterranean diet improves hepatic steatosis and insulin sensitivity in individuals with non-alcoholic fatty liver disease. J Hepatol, 59:138–43. 23485520 10.1016/j.jhep.2013.02.012

[56] D’Souza A M Beaudry JL Szigiato AA (2012). Consumption of a high-fat diet rapidly exacerbates the development of fatty liver disease that occurs with chronically elevated glucocorticoids. Am J Physiol Gastrointest Liver Physiol, 302:G850–63. 22268100 10.1152/ajpgi.00378.2011

[57] Mohanty SR Troy TN Huo D (2009). Influence of ethnicity on histological differences in non-alcoholic fatty liver disease. J Hepatol,50:797–804. 19231016 10.1016/j.jhep.2008.11.017

